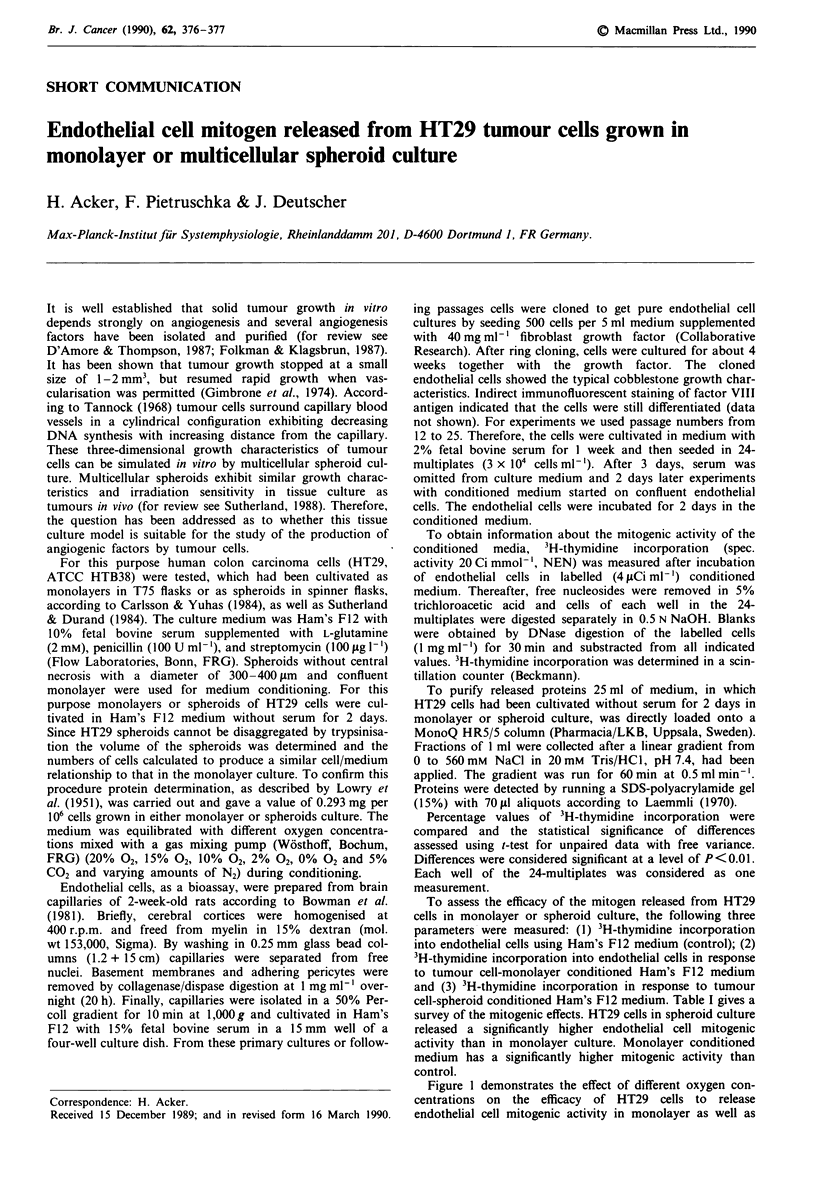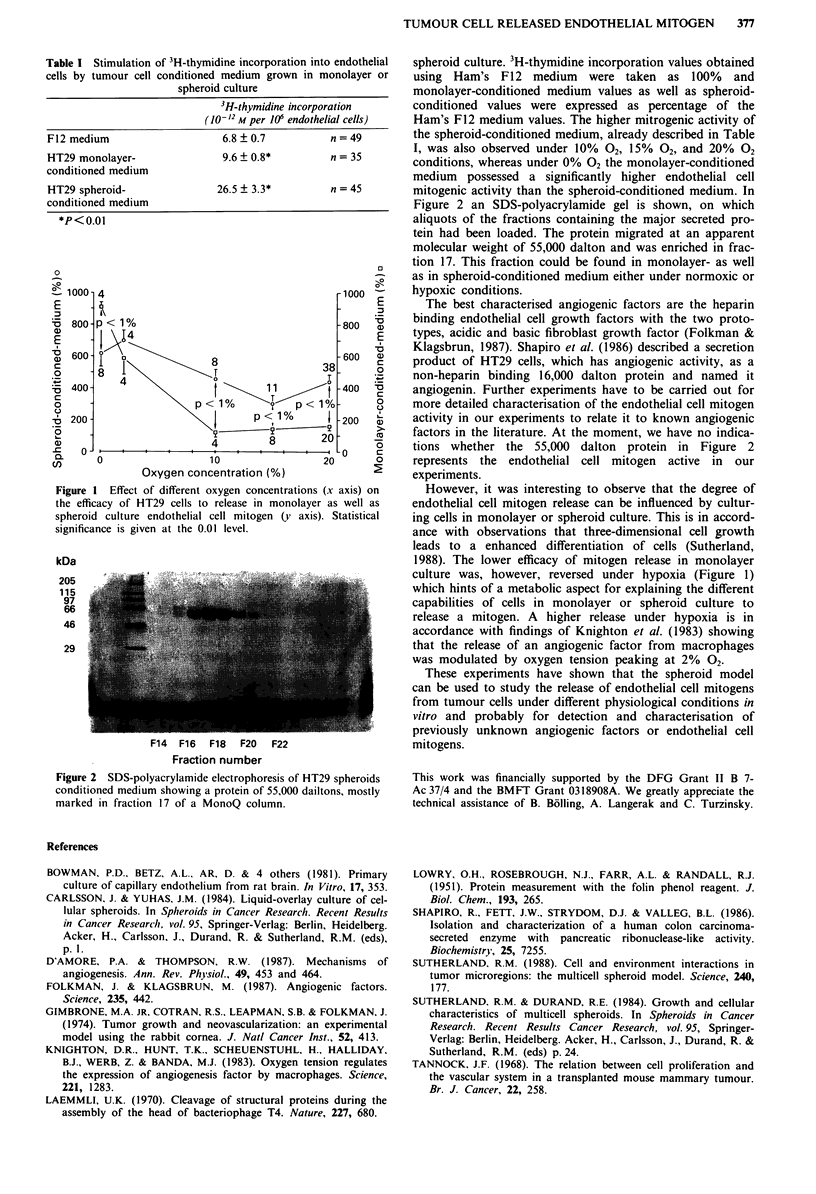# Endothelial cell mitogen released from HT29 tumour cells grown in monolayer or multicellular spheroid culture.

**DOI:** 10.1038/bjc.1990.301

**Published:** 1990-09

**Authors:** H. Acker, F. Pietruschka, J. Deutscher

**Affiliations:** Max-Planck-Institut für Systemphysiologie, Dortmund, FR Germany.

## Abstract

**Images:**


 © Macmillan Press Ltd., 1990

SHORT COMMUNICATION

### Endothelial cell mitogen released from HT29 tumour cells grown in monolayer or multicellular spheroid culture

H. Acker, F. Pietruschka & J. Deutscher

*Max-Planck-Institut für Systemphysiologie, Rheinlanddamm 201, D-4600 Dortmund 1, FR Germany.*

It is well established that solid tumour growth *in vitro* depends strongly on angiogenesis and several angiogenesis factors have been isolated and purified (for review see D'Amore & Thompson, 1987; Folkman & Klagsbrun, 1987). It has been shown that tumour growth stopped at a small size of 1–2 $mm^3$, but resumed rapid growth when vascularisation was permitted (Gimbrone *et al.*, 1974). According to Tannock (1968) tumour cells surround capillary blood vessels in a cylindrical configuration exhibiting decreasing DNA synthesis with increasing distance from the capillary. These three-dimensional growth characteristics of tumour cells can be simulated *in vitro* by multicellular spheroid culture. Multicellular spheroids exhibit similar growth characteristics and irradiation sensitivity in tissue culture as tumours *in vivo* (for review see Sutherland, 1988). Therefore, the question has been addressed as to whether this tissue culture model is suitable for the study of the production of angiogenic factors by tumour cells.

For this purpose human colon carcinoma cells (HT29, ATCC HTB38) were tested, which had been cultivated as monolayers in T75 flasks or as spheroids in spinner flasks, according to Carlsson & Yuhas (1984), as well as Sutherland & Durand (1984). The culture medium was Ham's F12 with 10% fetal bovine serum supplemented with L-glutamine (2 mM), penicillin (100 U $ml^{-1}$), and streptomycin (100 $\mu g\,l^{-1}$) (Flow Laboratories, Bonn, FRG). Spheroids without central necrosis with a diameter of 300–400 $\mu m$ and confluent monolayer were used for medium conditioning. For this purpose monolayers or spheroids of HT29 cells were cultivated in Ham's F12 medium without serum for 2 days. Since HT29 spheroids cannot be disaggregated by trypsinisation the volume of the spheroids was determined and the numbers of cells calculated to produce a similar cell/medium relationship to that in the monolayer culture. To confirm this procedure protein determination, as described by Lowry *et al.* (1951), was carried out and gave a value of 0.293 mg per $10^6$ cells grown in either monolayer or spheroids culture. The medium was equilibrated with different oxygen concentrations mixed with a gas mixing pump (Wösthoff, Bochum, FRG) (20% $O_2$, 15% $O_2$, 10% $O_2$, 2% $O_2$, 0% $O_2$ and 5% $CO_2$ and varying amounts of $N_2$) during conditioning.

Endothelial cells, as a bioassay, were prepared from brain capillaries of 2-week-old rats according to Bowman *et al.* (1981). Briefly, cerebral cortices were homogenised at 400 r.p.m. and freed from myelin in 15% dextran (mol. wt 153,000, Sigma). By washing in 0.25 mm glass bead columns (1.2 + 15 cm) capillaries were separated from free nuclei. Basement membranes and adhering pericytes were removed by collagenase/dispase digestion at 1 mg $ml^{-1}$ overnight (20 h). Finally, capillaries were isolated in a 50% Percoll gradient for 10 min at 1,000 *g* and cultivated in Ham's F12 with 15% fetal bovine serum in a 15 mm well of a four-well culture dish. From these primary cultures or following passages cells were cloned to get pure endothelial cell cultures by seeding 500 cells per 5 ml medium supplemented with 40 mg $ml^{-1}$ fibroblast growth factor (Collaborative Research). After ring cloning, cells were cultured for about 4 weeks together with the growth factor. The cloned endothelial cells showed the typical cobblestone growth characteristics. Indirect immunofluorescent staining of factor VIII antigen indicated that the cells were still differentiated (data not shown). For experiments we used passage numbers from 12 to 25. Therefore, the cells were cultivated in medium with 2% fetal bovine serum for 1 week and then seeded in 24-multiplates ($3 \times 10^4$ cells $ml^{-1}$). After 3 days, serum was omitted from culture medium and 2 days later experiments with conditioned medium started on confluent endothelial cells. The endothelial cells were incubated for 2 days in the conditioned medium.

To obtain information about the mitogenic activity of the conditioned media, $^3$H-thymidine incorporation (spec. activity 20 Ci $mmol^{-1}$, NEN) was measured after incubation of endothelial cells in labelled (4 $\mu Ci\,ml^{-1}$) conditioned medium. Thereafter, free nucleosides were removed in 5% trichloroacetic acid and cells of each well in the 24-multiplates were digested separately in 0.5 N NaOH. Blanks were obtained by DNase digestion of the labelled cells (1 mg $ml^{-1}$) for 30 min and substracted from all indicated values. $^3$H-thymidine incorporation was determined in a scintillation counter (Beckmann).

To purify released proteins 25 ml of medium, in which HT29 cells had been cultivated without serum for 2 days in monolayer or spheroid culture, was directly loaded onto a MonoQ HR5/5 column (Pharmacia/LKB, Uppsala, Sweden). Fractions of 1 ml were collected after a linear gradient from 0 to 560 mM NaCl in 20 mM Tris/HCl, pH 7.4, had been applied. The gradient was run for 60 min at 0.5 ml $min^{-1}$. Proteins were detected by running a SDS-polyacrylamide gel (15%) with 70 $\mu l$ aliquots according to Laemmli (1970).

Percentage values of $^3$H-thymidine incorporation were compared and the statistical significance of differences assessed using *t*-test for unpaired data with free variance. Differences were considered significant at a level of $P < 0.01$. Each well of the 24-multiplates was considered as one measurement.

To assess the efficacy of the mitogen released from HT29 cells in monolayer or spheroid culture, the following three parameters were measured: (1) $^3$H-thymidine incorporation into endothelial cells using Ham's F12 medium (control); (2) $^3$H-thymidine incorporation into endothelial cells in response to tumour cell-monolayer conditioned Ham's F12 medium and (3) $^3$H-thymidine incorporation in response to tumour cell-spheroid conditioned Ham's F12 medium. Table I gives a survey of the mitogenic effects. HT29 cells in spheroid culture released a significantly higher endothelial cell mitogenic activity than in monolayer culture. Monolayer conditioned medium has a significantly higher mitogenic activity than control.

Figure 1 demonstrates the effect of different oxygen concentrations on the efficacy of HT29 cells to release endothelial cell mitogenic activity in monolayer as well as

Correspondence: H. Acker.
Received 15 December 1989; and in revised form 16 March 1990.

**Table I** Stimulation of $^{3}$H-thymidine incorporation into endothelial cells by tumour cell conditioned medium grown in monolayer or spheroid culture

| | *$^{3}$H-thymidine incorporation ($10^{-12}$ M per $10^{6}$ endothelial cells)* | |
|---|---|---|
| F12 medium | 6.8 ± 0.7 | $n = 49$ |
| HT29 monolayer-conditioned medium | 9.6 ± 0.8* | $n = 35$ |
| HT29 spheroid-conditioned medium | 26.5 ± 3.3* | $n = 45$ |

*$P$<0.01

**Figure 1** Effect of different oxygen concentrations ($x$ axis) on the efficacy of HT29 cells to release in monolayer as well as spheroid culture endothelial cell mitogen ($y$ axis). Statistical significance is given at the 0.01 level.

**Figure 2** SDS-polyacrylamide electrophoresis of HT29 spheroids conditioned medium showing a protein of 55,000 dailtons, mostly marked in fraction 17 of a MonoQ column.

spheroid culture. $^{3}$H-thymidine incorporation values obtained using Ham's F12 medium were taken as 100% and monolayer-conditioned medium values as well as spheroid-conditioned values were expressed as percentage of the Ham's F12 medium values. The higher mitrogenic activity of the spheroid-conditioned medium, already described in Table I, was also observed under 10% $O_2$, 15% $O_2$, and 20% $O_2$ conditions, whereas under 0% $O_2$ the monolayer-conditioned medium possessed a significantly higher endothelial cell mitogenic activity than the spheroid-conditioned medium. In Figure 2 an SDS-polyacrylamide gel is shown, on which aliquots of the fractions containing the major secreted protein had been loaded. The protein migrated at an apparent molecular weight of 55,000 dalton and was enriched in fraction 17. This fraction could be found in monolayer- as well as in spheroid-conditioned medium either under normoxic or hypoxic conditions.

The best characterised angiogenic factors are the heparin binding endothelial cell growth factors with the two prototypes, acidic and basic fibroblast growth factor (Folkman & Klagsbrun, 1987). Shapiro *et al.* (1986) described a secretion product of HT29 cells, which has angiogenic activity, as a non-heparin binding 16,000 dalton protein and named it angiogenin. Further experiments have to be carried out for more detailed characterisation of the endothelial cell mitogen activity in our experiments to relate it to known angiogenic factors in the literature. At the moment, we have no indications whether the 55,000 dalton protein in Figure 2 represents the endothelial cell mitogen active in our experiments.

However, it was interesting to observe that the degree of endothelial cell mitogen release can be influenced by culturing cells in monolayer or spheroid culture. This is in accordance with observations that three-dimensional cell growth leads to a enhanced differentiation of cells (Sutherland, 1988). The lower efficacy of mitogen release in monolayer culture was, however, reversed under hypoxia (Figure 1) which hints of a metabolic aspect for explaining the different capabilities of cells in monolayer or spheroid culture to release a mitogen. A higher release under hypoxia is in accordance with findings of Knighton *et al.* (1983) showing that the release of an angiogenic factor from macrophages was modulated by oxygen tension peaking at 2% $O_2$.

These experiments have shown that the spheroid model can be used to study the release of endothelial cell mitogens from tumour cells under different physiological conditions *in vitro* and probably for detection and characterisation of previously unknown angiogenic factors or endothelial cell mitogens.

This work was financially supported by the DFG Grant II B 7-Ac 37/4 and the BMFT Grant 0318908A. We greatly appreciate the technical assistance of B. Bölling, A. Langerak and C. Turzinsky.

### References

BOWMAN, P.D., BETZ, A.L., AR, D. & 4 others (1981). Primary culture of capillary endothelium from rat brain. *In Vitro*, **17,** 353.

CARLSSON, J. & YUHAS, J.M. (1984). Liquid-overlay culture of cellular spheroids. In *Spheroids in Cancer Research. Recent Results in Cancer Research, vol. 95*, Springer-Verlag: Berlin, Heidelberg. Acker, H., Carlsson, J., Durand, R. & Sutherland, R.M. (eds), p. 1.

D'AMORE, P.A. & THOMPSON, R.W. (1987). Mechanisms of angiogenesis. *Ann. Rev. Physiol.*, **49,** 453 and 464.

FOLKMAN, J. & KLAGSBRUN, M. (1987). Angiogenic factors. *Science*, **235,** 442.

GIMBRONE, M.A. JR, COTRAN, R.S., LEAPMAN, S.B. & FOLKMAN, J. (1974). Tumor growth and neovascularization: an experimental model using the rabbit cornea. *J. Natl Cancer Inst.*, **52,** 413.

KNIGHTON, D.R., HUNT, T.K., SCHEUENSTUHL, H., HALLIDAY, B.J., WERB, Z. & BANDA, M.J. (1983). Oxygen tension regulates the expression of angiogenesis factor by macrophages. *Science*, **221,** 1283.

LAEMMLI, U.K. (1970). Cleavage of structural proteins during the assembly of the head of bacteriophage T4. *Nature*, **227,** 680.

LOWRY, O.H., ROSEBROUGH, N.J., FARR, A.L. & RANDALL, R.J. (1951). Protein measurement with the folin phenol reagent. *J. Biol. Chem.*, **193,** 265.

SHAPIRO, R., FETT, J.W., STRYDOM, D.J. & VALLEG, B.L. (1986). Isolation and characterization of a human colon carcinoma-secreted enzyme with pancreatic ribonuclease-like activity. *Biochemistry*, **25,** 7255.

SUTHERLAND, R.M. (1988). Cell and environment interactions in tumor microregions: the multicell spheroid model. *Science*, **240,** 177.

SUTHERLAND, R.M. & DURAND, R.E. (1984). Growth and cellular characteristics of multicell spheroids. In *Spheroids in Cancer Research. Recent Results Cancer Research, vol. 95*, Springer-Verlag: Berlin, Heidelberg. Acker, H., Carlsson, J., Durand, R. & Sutherland, R.M. (eds) p. 24.

TANNOCK, J.F. (1968). The relation between cell proliferation and the vascular system in a transplanted mouse mammary tumour. *Br. J. Cancer*, **22,** 258.